# Architecture of a Data Portal for Publishing and Delivering Open Data for Atmospheric Measurement

**DOI:** 10.3390/ijerph20075374

**Published:** 2023-04-03

**Authors:** Rosa Virginia Encinas Quille, Felipe Valencia de Almeida, Mauro Yuji Ohara, Pedro Luiz Pizzigatti Corrêa, Leandro Gomes de Freitas, Solange Nice Alves-Souza, Jorge Rady de Almeida, Maggie Davis, Giri Prakash

**Affiliations:** 1 School of Arts, Sciences and Humanities, University of São Paulo, Rua Arlindo Béttio, 1000-Ermelino Matarazzo, São Paulo 03828-000, Brazil; 2 Residues and Contaminated Areas Laboratory (LARC), Institute for Technological Research (IPT), Av. Prof. Almeida Prado, 532-Butantã, São Paulo 05508-901, Brazil; 3 Polytechnic School, University of São Paulo, Av. Prof. Luciano Gualberto, 380-Butantã, São Paulo 05508-010, Brazil; felipe.valencia.almeida@usp.br (F.V.d.A.);; 4 Environmental Sciences Division, Oak Ridge National Laboratory, 1 Bethel Valley Road, Oak Ridge, TN 37831, USA

**Keywords:** open data, atmospheric data measurement, data portal requirements, FAIR principles, open science, big data

## Abstract

Atmospheric data are collected by researchers every day. Campaigns such as GOAmazon 2014/2015 and the Amazon Tall Tower Observatory collect essential data on aerosols, gases, cloud properties, and meteorological parameters in the Brazilian Amazon basin. These data products provide insights and essential information for analyzing and predicting natural processes. However, in Brazil, it is estimated that more than 80% of the scientific data collected are not published due to the lack of web portals that collect and store these data. This makes it difficult, or even impossible, to access and integrate the data, which can result in the loss of significant amounts of information and significantly affect the understanding of the overall data. To address this problem, we propose a data portal architecture and open data deployment that enable Big Data processing, human interaction, and download-oriented approaches with tools that help users catalog, publish and visualize atmospheric data. Thus, we describe the architecture developed, based on the experience of the Atmospheric Radiation Measurement Data Center, which incorporates the principles of FAIR, the infrastructure and content management system for managing scientific data. The portal partial results were tested with environmental data from contaminated areas at the University of São Paulo. Overall, this data portal creates more shared knowledge about atmospheric processes by providing users with access to open environmental data.

## 1. Introduction

Both society and the environment have experienced significant impacts due to increasing climate change [1,2,3,4,5,6], leading to an environmental crisis [7]. This crisis is due to environmental damage caused mainly by humans and natural disasters [8,9,10,11,12,13]. The current environmental problems can be classified into key areas, such as air pollution, water pollution, soil pollution, water scarcity, biodiversity reduction, climate change and atmospheric problems. The scientific community is constantly researching these problems [14,15,16,17,18], and nature-based solutions, radical solutions and others have also been proposed [19,20,21,22]. Technology plays an essential role in all of them. Instruments that can measure environmental and atmospheric properties are commonplace; these data can be analyzed, and decisions can then be made based on the results found. The large amount of data collected brings new challenges for storing and protecting, and ensuring the integrity of data. Solutions to these challenges lie in Big Data processing, human interaction, and download-oriented approaches.

The scientific community has adopted open science initiatives worldwide for greater transparency, reliability, and replicability. For example, Allen et al. (2019) [23] presents three benefits and three challenges of open science, stating that, although the challenges requiring a change in attitude and expectations about productivity, it is worth adopting open methods to provide greater faith in research, create new helpful systems and invest in the future, as they are necessary and unavoidable. More recent studies, such Ramachandran et al. (2021) [24], define open science as “collaborative culture enabled by technology that empowers the open sharing of data, information, and knowledge within the scientific community and the wider public to accelerate scientific research and understanding”, for which the authors describe actions for an open science paradigm shift, highlighting the importance of data programs, open policy development, investment in innovative and collaborative infrastructures, and the promotion of cultural change. Open access databases and analysis tools promote open science in different study domains, which, in this context, are currently based on the FAIR (Findability, Accessibility, Interoperability, and Reuse) data principles [25,26]. Although these principles guide data management with standardized policies, they do not specify which technologies, tools and requirements to use in infrastructure and architecture for efficient data management.

This type of data management is based on a web platform, called a “Data Portal”. This portal must be easy to use for the management, visualization, analysis and interpretation of data at the Big Data level. Another complementary aspect that has gained importance is the incorporation of intelligence in these platforms. Several papers present techniques to make portals intelligent by using concepts, such as semantic web [27], Web intelligence [28,29], and Artificial Intelligence (AI) [30]. In addition, we should mention that monitoring and data management require real-time machine learning-enabled approaches to infrastructure and risk management [31,32]. Data publications are diverse in the research world. For example, Linked Data [33] is a framework for publishing open US government data and Monitor My Watershed [34] is a data portal for environmental monitoring. Wu et al. (2021) [35] proposed an open and interoperable climate data portal for Ireland and England, based on the following three components: (1) climate analysis ontology, (2) ad hocSPARQL server, and (3) dereferencing engine deployed to resolve URIs for entity information; and we present other data portals with similar purposes in Section 3.5, comparing them with our architecture proposal.

Some problems have been identified regarding a portal suitable for requirements to integrate web applications. One of the significant problems is simultaneous large requests to provide different types of information consistently from different vendors. Regarding performance, problems may arise due to numerous requests and excessive waiting times to process requests. The Atmospheric Radiation Measurement (ARM) Data Center provides an infrastructure for open access to multidimensional climate data and atmospheric observations derived from various global climate data. ARM archives more than 22 million user-accessible data files, stored primarily in the NetCDF file format, with a total data volume of nearly 3 petabytes [36].

The atmospheric data collected by the Brazilian research community come mainly from projects such as Green Ocean Amazon (GOAmazon 2014/15) [37], the Amazon Tall Tower Observatory [38], the Aerosols, Clouds, Convection Experiment (ACONVEX), Cloud Processes of the Main Precipitation Systems in Brazil, and other projects of the Atmospheric Physics Laboratory (LFA-USP). These research projects have national and international partnerships, including ARM, the Max Planck Institute, NASA, Harvard University, Stockholm University, Lille University, the National Institute for Amazon Research, the National Institute for Space Research, and several Brazilian universities. Recent studies indicate that there is a lack of maturity models for managing the data [39]. Maturity models, in our context, refer to the ways and efforts involved in establishing quality management at all levels of the architecture. One of the cases in Brazil is the environmental monitoring of the contaminated areas on the Campus of the School of Arts, Sciences and Humanities of the University of São Paulo (EACH-USP). We observed that, despite the richness of datasets, there are management and availability problems, confirming the opportunity to carry out proofs of concept with the methodology proposed.

This paper presents a data portal architecture and its partial results from the EACH-USP case to publish and provide open data for environmental measurements based on the ARM portal architecture. With this proposal, we answer the question: why is developing an open data portal architecture with Big Data processing capacity important? Categories are defined for data publication, such as queries, specialized queries, result presentations, data exploration and data services.

## 2. Environmental Data Measurement as a Big Data Problem: EACH-USP Case Study

In order to understand and predict the behavior of environmental phenomena on Earth, a complex analysis process is required. This process begins with observing components measured by instruments from weather/climate systems. Observations are collections of countless interacting molecules, and their quantification can be called atmospheric/land/ocean/ice data measurement. These measurements include mumerous variables, such as sea level pressure, wind field, ozone concentration, air temperature, pressure and considerably more.

The rapid advancement of technologies brings significant advantages to environmental monitoring. For example:

- Electronic miniaturization, which allows sustainability (low energy consumption and low cost);
- Instrument sensitivity, increasing precision, resolution and data coverage;
- Distribution and ubiquity of sensors.

**Figure 1:**
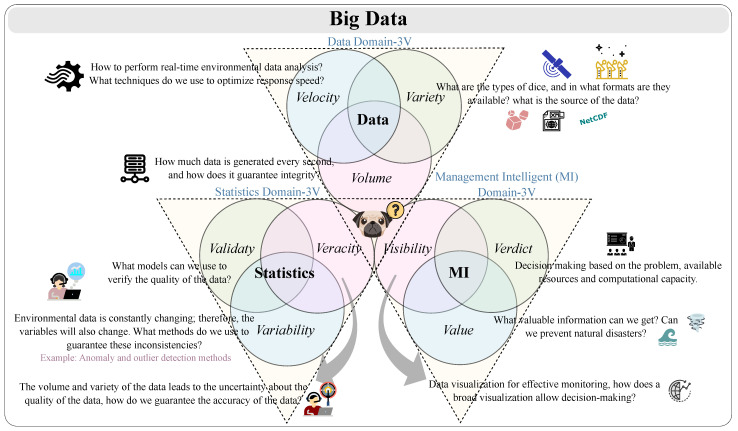
Perspectives of challenges presented in a Big Data context for environmental monitoring.

However, with these advances, challenges are detected in the Big Data area, such as the fact that, over time, the records have increased, leading to immense data volume growth (scale of data). The increase in temporal resolution demands higher data velocity (the analysis of streaming data). Furthermore, instruments increase the variety of data (different forms of data). Volume, velocity and variety were originally known as the 3 Vs of Big Data. We can also analyze the 3 Vs in different domains. We are hence facing 9 Vs in Big Data ($3^{2}$ Vs), the classification of which is based on the perspectives defined by Caesar et al. [40]. In Figure 1, we illustrate these concerns.

Environmental data is generated and stored in various types of formats. NetCDF is a scientific data storage format that forms a set of interfaces for accessing array-oriented data. NetCDF organizes data into variable, dimension, attribute, and group definitions, facilitating access for their management.

**Figure 2:**
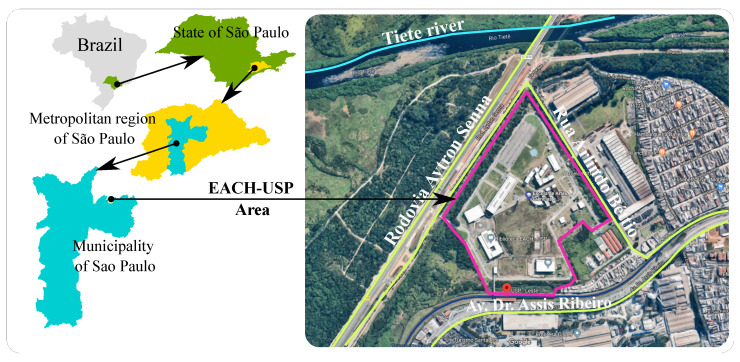
Location of the study area (Source: Adapted from Google Maps).

As an example of an environmental data measurement problem, we present the School of Arts, Sciences and Humanities of the University of São Paulo (EACH-USP) case of contaminated areasmonitoring. Figure 2 shows the EACH-USP study area. This area is part of the registry of contaminated areas of the state of São Paulo, according to the Environmental Company of the State of São Paulo (CETESP) [41].

Studies investigated the occurrence of methane gas in the area, originating from the organic matter present in the layers of anthropic origin, derived from the dredging of the Tietê River, and in the natural layers belonging to the quaternary alluvial deposits, also associated with the Tietê River. The results of the studies led to the belief that the predominant chemical compound in the gaseous atmosphere of the soil pores in the area is methane gas, with a less frequent occurrence of volatile organic vapors. Due to this occurrence of gases, a ventilation system was installed in the gravel mats, aiming to prevent entry of gas into the buildings. This ventilation system was not implemented to remediate the soil but to keep the carpet ventilated, and to prevent the accumulation and intrusion of gases into the buildings. The main action of this implementation, which began in March 2014, is the systematic monitoring of methane gas and other volatile organic compounds in the soil and the ventilation systems under the buildings [42].

In the context presented, the main environmental monitoring variables at EACH-USP are the concentrations of gases and vapors in the subsoil. In addition, several other correlated variables also have regularly been monitored. They are of great importance to this experiment, including atmospheric variables (i.e., temperature, pressure and rainfall), physical-chemical variables (i.e., pH and dissolved oxygen in groundwater) and hydrogeological variables (i.e., groundwater level). These data are publicly accessible on the Superintendence of Physical Space page at USP [43].

In 2014, the first campaign was conducted to install Soil Gas Monitoring Wells (SGMWs), resulting in a regular historical series up to the present. Currently, 115 sets of SGMWs are being monitored, in which measurements are taken at two different depths in 104 wells and at three depths in nine other sets, totaling 236 sampling points, sampled weekly [42]. In addition to these wells, 173 points of infrastructure in the buildings are monitored fortnightly (i.e., drains, passage boxes) and weekly in 22 exhaust fans present in the ventilation systems of the buildings [42]. For this monitoring, two portable pieces of equipment are used, the GEM 5000, to measures gas concentrations and parameters related to the migration of biogas in the soil, which includes methane gas; and the MX6 equipment, to measure the concentration of VOCs, besides quantifying the flammability of gases through the “Lower Flammability Limit” (LEL).

In addition to regular monitoring, the history of environmental studies at EACH-USP includes a vast amount of reports issued by consulting companies and public bodies, allowing access to a vast collection of important metadata to understand the phenomena and dynamics of migration of gases in soil. Examples of these data can be listed as follows: results of chemical analyses of soil and groundwater, lithological profiles of drillings carried out, mathematical models and technical opinions. In addition to these reports, it is also considered essential to supplement the information with other data sources, such as climate data provided by meteorological companies.

**Figure 3:**
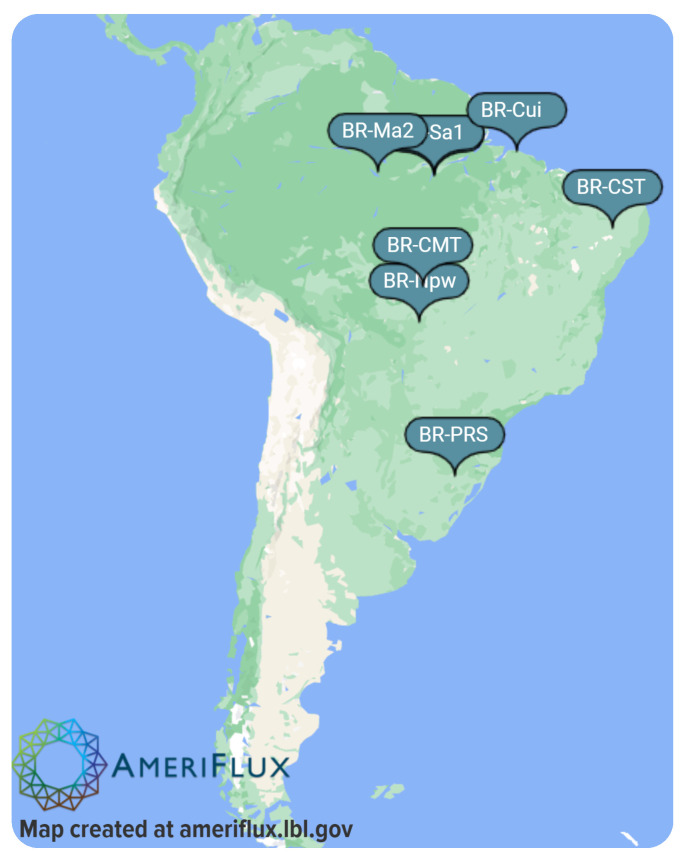
AmeriFlux measurement towers in Brazil.

After defining the EACH-USP project, a more general overview of environmental measurement problems in Brazil is necessary. Brazil has numerous measurement towers, which collect different environmental data in different places on its territory. For example, Figure 3 presents only a few towers from the AmeriFlux project [44].

When considering the myriad of different towers related to various projects, each tower can publish its measured data in a separate repository. This hinders access to their data. A central data portal would be an alternative to make this job more manageable. The following section discusses some aspects of the Data Portal Design.

## 3. Data Portal Design

Data portals have been created for specific purposes, as in the Brazilian Biodiversity Information System (SiBBr) case. This portal was based on the architecture of the Atlas of Living Australia (ALA) [45,46,47]. It was developed as an information system to integrate and disseminate data collected and published by different Brazilian institutions (universities, research institutes and government agencies). Global Biodiversity Information Facility (GBIF) [48,49] is an example of an international data portal.

Building the data portal is a prime consideration in data publishing and delivery systems. These systems have specific requirements before their construction, such as the definition of a data quality framework (data quality), metrics conventions for efficient handling of data (metrics) and web services for data distribution (monitoring). Implementing these requirements can allow Big Data processing through a scalable architecture with faster data access and fewer data movements, creating valuable and intuitive end-user tools that enable human interaction and download-oriented approaches in different formats that can be optimally reused. We thus address the following issues:

- FAIR principles
- FAIR tools adapted for Brazilian data portal
- Data portal architecture
- Analysis and data management
- Related data portals

### 3.1. FAIR Principles

FAIR (Findability, Accessibility, Interoperability, and Reusability) can be described as a series of principles to optimize the reuse of data [50].

Findability is related to how easy the data can be found. For example, if the data portal has rich metadata with all the available data, researchers can quickly verify if that data is helpful to them. It is also possible to locate the data quickly with search engines. Accessibility describes how easy it is to access the data after locating it. Using standardized protocols, such as the File Transfer Protocol (FTP), can help to achieve good accessibility. Interoperability is the capacity to integrate some data with other data from a different source. This is very important, since numerous Data Science experiments use data from many different sources. One way to achieve good interoperability is to use standardized data formats, such as the NetCDF. Finally, Reusability is related to the primary purpose of the FAIR principles. Data license and provenance are essential factors to help with data reusability.

Each of these principles is divided into topics, which are presented next.

1. Findability Principle
  F1: (meta)data are assigned a globally unique and persistent identifier
  F2: data are described with rich metadata (defined by R1below)
  F3: metadata clearly and explicitly include the identifier of the data described
  F4: (meta)data are registered or indexed in a searchable resource
2. Accessibility Principle
  A1: (meta)data are retrievable by their identifier using a standardized communications protocol
  A1.1: the protocol is open, free, and universally implementable
  A1.2: the protocol allows for an authentication and authorization procedure, where necessary
  A2: metadata are accessible, even when the data are no longer available
3. Interoperability Principle
  I1: (meta)data use a formal, accessible, shared, and broadly applicable language for knowledge representation
  I2: (meta)data use vocabularies that follow FAIR principles
  I3: (meta)data include qualified references to other (meta)data
4. Reusability Principle
  R1: meta(data) are richly described with a plurality of accurate and relevant attributes
  R1.1: (meta)data are released with a clear and accessible data usage license
  R1.2: (meta)data are associated with detailed provenance
  R1.3: (meta)data meet domain-relevant community standards

The domain must be considered to implement and interpret these principles, since the FAIR principles do not specify technologies and techniques for their implementation. According to Jacobsen et al. (2020) [51], various interpretations and implementations can create particular solutions for each case, which can be adapted over time as technology advances. Scientific communities have conducted studies to guide implementations [52,53,54,55,56,57]. Other research, related to the use of the FAIR principles in the environmental context, include Kinkade et al. (2022) [58], who specifically discuss the domain of geosciences, and Sarramia et al. (2022) [59], describing a Data Lake Architecture.

The FAIR principles serve as the basis for constructing the data portal. Since they are recognized as fundamental by the Data Science community, decisions were taken to meet these principles during the project development.

### 3.2. FAIR Tools Adopted for Brazilian Data Portal

Our data portal architecture proposal should provide comprehensive capabilities for Brazilian multidimensional environmental data, including storage, management and data distribution. These capabilities have (functional) requirements and processing to carry out queries, specialized queries, presentation of results, data export and data services. In the V (2020) [60] and VI (2022) [61] Data Science Workshop, in collaboration with different Brazilian institutions and the ARM data center, data analysis and management methodologies to put into practice were discussed. The application of open science and the use of FAIR tools in Big Data were discussion topics. The adopted tools answer the following questions:

(1) How to deal with open and closed (publishable) data?
  Dealing with open data and closed data as needed is still a challenge. In Brazil, open science has been promoted through data portals in recent years. Such is the case for INPE, which has been carrying out space studies since 1971 and is considered one of the first to offer information on its space observations with open science practice initiatives [62]. The INPE catalog contains historical data since 1973, which allows monitoring of environmental, urban and water changes. Since 2004, INPE has made terrestrial resources available on its data portal (TerraAmazon). However, it faces data availability and political challenges as it is generally quarterly. Their main hurdle is making the data accessible and understandable to the general public.
  Meanwhile, the Institute for Technological Research of the State of São Paulo (IPT) has offered technological solutions to industry, and public policies, since its inception in 1899. The IPT has laboratories for environmental studies of the following: water, contaminated areas, forests, environmental management, air quality, waste, and sustainability. For example, the contaminated areas laboratory has data acquisition and treatment for intervention plans. Managing contaminated areas became part of the agenda to mitigate environmental impacts. The IPT considers managing contaminated areas one of the most significant challenges for regulatory bodies, entrepreneurs, academics, professionals, and society. In recent years, they have been promoting open science, through projects such as PDIp (Institutional development plan, in the area of digital transformation, and advanced manufacturing and smart and sustainable cities (PDIp)), for open data through a data portal. As an institution that works with a wide variety of institutions and companies, IPT is, to some extent, dealing with closed data, with prospects of being made available in an open way.
  The importance of the practice of open science is evident in the fact that it brings benefits of accessibility and collaboration. To deal with open and closed data (protection of confidential data), we propose tools designed with FAIR principles.
(2) What is the size or our problem?
  The size of our problem is determined according to the challenges posed by Big Data research for environmental data, as discussed in Section 2. Working with Big Data from the user’s computer is not feasible. Cloud services can be a solution for the development of the required tools.
(3) What are the atomic parts of the problem?
  In the architecture of the Big Data portal, there are three levels (Infrastructure, FAIR tools and Applications), which we can consider as types of problems. Each one of them divides into atomic parts of a problem which are addressed.
(4) Who are the users?
  We classify users into four types: the scientific community (researchers, academics, and others.), analysts (environmental data analysts, specialists, and others.), decision-makers (institutions such as CETESB, managers, and others.) and general. Permissions are provided for certain functionalities depending on classification and registration authentication. For example, suppose there were a case of closed data where privacy, security or confidentiality terms were a limitation for the data to be found for the general public. In this case, the data could be available to other types of users with greater rigor of registration.
(5) What is the impact on society?
  The practice of open science in the environmental area can impact society by addressing environmental problems and, consequently, social problems. Research institutions, governments and organizations can make better decisions. An efficient data portal can support the development of solutions, and answer questions, such as the following: What are the reactive chemical substances destroying the ecosystem and what can be done about them?; How do we reduce soil contamination?; What remediation techniques do we apply to a specific problem in a contaminated area?

To address these issues, the proposal is based on ARM’s FAIR tools that support the data life cycle. This life cycle continuously improves the quality and delivery of data products to the end user.

### 3.3. Data Portal Architecture

**Figure 4:**
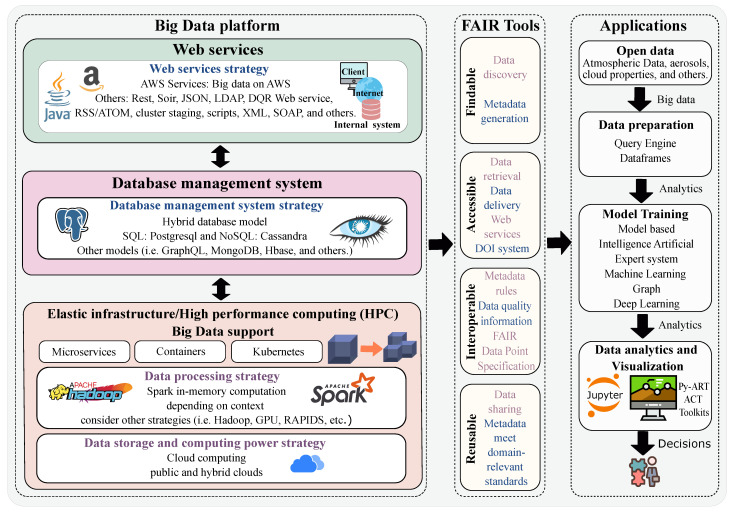
Overall view of the architecture of the data portal.

Data portal architecture is associated with technological changes that enable people to generate, store, retrieve, and analyze large amounts of data. In Figure 4, we present an architecture that supports essential requirements in building the data portal. These requirements include FAIR tools for data analysis and management. These tools efficiently help the interaction of different end users (data analysts, data scientists, and the scientific community).

The data portal is executed under an elastic infrastructure for data processing with Big Data level support. The elasticity consists of considering storage strategies and computational power, which, depending on the amount and complexity of the data, can be processed in a public or hybrid cloud. Data processing strategies rely on the context, as data can be processed in memory (Spark), on disk (Hadoop) or in other ways (e.g., GPU and RAPIDs). The architectural design considers a distributed application to obtain a scalable implementation in which an application can split can be split into independent services (microservices). We, therefore, guarantee that each service can be extended or updated without interrupting the execution of other services in the application. For standalone execution in a lightweight, efficient and standardized way we can use containers (e.g., Kubernetes).

The database management system is a hybrid model, yet we also consider NoSQL (non-relational model) as it uses a distributed architecture, wherein data is kept on multiple servers. The system, thus, allows scalability by adding more servers as needed. With this model, server failure is no longer a problem and can be tolerated. SQL (relational model) and others (e.g., GraphQL and Hbase) are considered to develop some FAIR tools. As web service strategies, architectures can be designed over AWS services as they provide aspects of Big Data technology (e.g., Amazon Kinesis, AWS Lambda, DynamoDB and EC2). Other services, such as Rest, Soir, JSON, LDAP and DQR Web Services, can be used.

In developing scientific data management and administration tools, the principles of FAIR are considered. FAIR maintains good practices for publishing scientific data. Data and metadata can be found using search tools. These tools include data discovery systems, metadata services, DOI systems, Online Metadata Editor (OME), DQ Tools, user registration tools, data storage and distribution.

The architecture foresees the final applications which enable the publication and open provision of atmospheric data, data preparation through query engines and dataframes, training of models that can be artificially intelligent, and data analysis and visualization systems through APIs.

### 3.4. Analysis and Data Management

The method simplifies the two-data life-cycle, consolidating each stage of the DataONE life-cycle [63] into a more straightforward data management cycle. To achieve our goal, we needed to develop the following main components for publishing and making data for aerosol measurements openly available: implementation details (overall workflow), metadata creation (Online Metadata Editor [OME]), data discovery, data citation, and data sharing among portals using standards and protocols.

**Figure 5:**
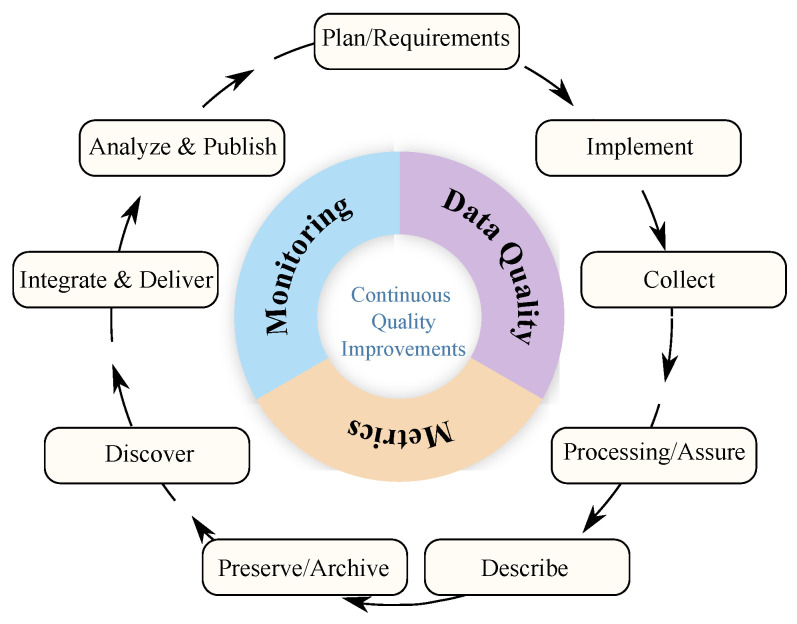
ARM adapted Data management life-cycle.

**Figure 6:**
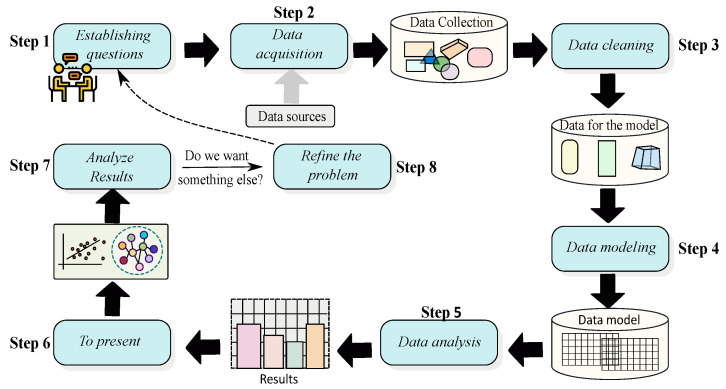
Analysis process for data science.

Figure 5 depicts the data life-cycle for management, whereby each state can be considered a data quality, metric, or monitoring tool. In Figure 6, the process of data analysis within data science is depicted.

### 3.5. Related Data Portals

This section presents a comparison table (see Table 1) of environmental data portals. The comparison was made based on the FAIR principles presented in Section 3.1.

INPE, a Brazilian institution linked to the Ministry of Science, Technology and Innovations, promotes the opening of data from its Remote System Datacenter (CDSR). It is responsible for the reception, processing and distribution of images acquired by satellites (for example, AMAZONIA-1, CBERS-04A, LANDSAT-7, LANDSAT-8, TERRA, S-NPP, GOES-16 and MetOp-B). Although, in 2019, the “Brazilian Data Cube” project began for the entire national territory to deal with large volumes of data, the challenges that a Big Data infrastructure demands are still under development. On the other hand, even though they do not stipulate FAIR principles for their tools, we can identify the equivalence of some principles, as shown in the comparison table. At the level of Brazilian portals, it has been promoting the practice of open science the most through its data inventory work plan, metadata cataloging, and implementation of the CKAN–INPE data management platform.

SiBBr is a Brazilian national data portal that helps ensure data-driven policy and design by integrating information on biodiversity. SiBBr has a collaborative network of institutions and actors, currently integrating more than 500 data sets from more than 160 publishers that share more than 23 million records. Even though the portal has the potential to be an integrating portal, due to the variety of collaborators, it does not present an architecture by the FAIR principles for Big Data. This is the second Brazilian portal that promotes data publication for free, contributing to open science.

GBIF, ALA, LINCS, Linked Open Data, and ARM are international portals using FAIR principles to manage their data domain. They differ in their data domain, so their tools vary. On the other hand, Linked Open Data practices principles based on 5-stars and was included in the table according to its equivalence with the FAIR principles. Note that, regarding research in Big Data, not all have come to be mature in regard to these challenges.

Among the data portals presented, INPE and SiBBr do not present a portal based on FAIR principles but have minimal FAIR tools. On the contrary, the international portal, Linked Open Data has a more focused design with FAIR principles. In this case, it still needs to present Big Data approaches. However, ALA, GBIF, and LINCS, due to the volume of data, are beginning to partially insert Big Data concepts. The ARM portal is a closer example of inserting FAIR and Big Data principles into all data analysis and management methodology stages. We propose, in our architecture, to improve access to scientific data in Brazil through these methodologies and principles tested in other portals.

When comparing our proposed architecture with the ARM Data Portal, the main differences in our proposal arethe following: (1) computational infrastructure, (2) data domain, and (3) Big Data from a 9V perspective. In regard to the first difference, ARM works out of Oak Ridge National Laboratory (ORNL), where the world’s two largest supercomputers are currently located, these being the Frontier supercomputer, with an 1.1 exaflops system, and the Summit supercomputer, with a 200 petaflops system, demonstrating that Big Data experiments can reach their maximum capacity. Brazil does not have supercomputers of this magnitude. Due to this difference, we included flexibility concepts in the different layers of the architecture, such as the use of Hybrid Cloud Computing. The second difference is in the data domain. ARM has monitoring instruments in different parts of the planet by air, land, and water. Even though projects are underway for this in Brazil, Brazil still needs to include specific monitoring. However, Brazil has rich and vital monitoring sources, such as the INPE Monitoring Instrumentation, IPT, IFUSP, and others. Finally, the third difference is in classifying the study with Big Data, explained in Section 2.

## 4. ARM Data Portal

ARM is an U.S. Department of Energy scientific user facility with multiple laboratories and is a significant contributor to national and international climate research efforts. ARM provides diverse and comprehensive measurements from three highly instrumented, fixed ground sites on the North Slope of Alaska, the southern Great Plains, and the eastern North Atlantic. In addition, ARM operates and maintains an aerial facility and several mobile facilities. The ARM Data Center at the U.S. Department of Energy’s Oak Ridge National Laboratory accounts for providing end-to-end data services for multidimensional climate data, including data storage, management, and distribution. This section discusses several new and improved data and metadata tools recently developed by the ARM Data Center. These tools are used primarily by atmospheric scientists to perform a variety of tasks, including metadata management (http://adc.arm.gov/MetadataService, http://adc.arm.gov/armome, accessed on 10 January 2022), data discovery (https://adc.arm.gov/discovery, accessed on 10 January 2022), data citation (https://adc.arm.gov/armdoi, accessed on 10 January 2022), data access via web services (https://adc.arm.gov/armlive, accessed on 10 January 2022), and data quality reporting (https://adc.arm.gov/DQPRSearch, accessed on 10 January 2022). It has a microservices software development architecture with reusable components, such as a front-end UI/form (to collect information entered by the user), and an API (that accepts HTTP/S requests either via a UI-form or a command call such as curl and wget), and the database.

### 4.1. Metadata Service

**Figure 7:**
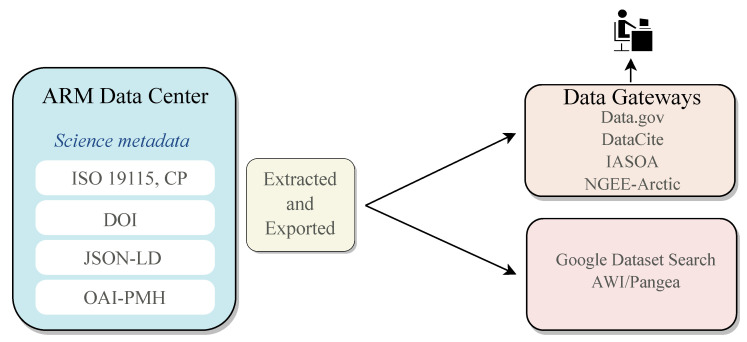
Meta sharing in external portals (Source: ARM).

ARM introduces a standards-based strategy for metadata management involving experts in the field. For its efficiency, it has an evolutionary perspective, with data quality checks, very detailed descriptions, and consideration of multiple data sources and data types. It allows data preservation and dissemination through this metadata system. The metadata system includes data processing and quality, temporal or spatial data, instrument information, and variables. Figure 7 shows the sharing process in external portals.

### 4.2. Online Metadata Editor (OME)

Scientific data often comes with complex and diverse metadata critical for data discovery and for users. The OME tool, developed at Oak Ridge National Laboratory for ARM, effectively manages various scientific datasets via ARM. OME is a standards-based tool that allows scientists to create and maintain metadata about their data products. It has been improved with new workflows that help metadata coordinators and researchers to submit and review their data more efficiently. Researchers use OME to enter relevant metadata into a web-based form. From the form, OME can create an XML file on the server where the editor is installed or on the user’s computer. Researchers can also use OME to modify existing metadata files. OME enables big data centers, such as ARM, to create meaningful, high-quality, standards-based descriptive information about their data products, making the data easier to find.

### 4.3. Data Discovery

The architecture of the data discovery system includes three main components: a metadata extractor, which extracts discovery-level metadata from data file headers and databases; an indexing system, based on Solr 8.0, that can create the distributed search index for the millions of data files; and a recently redesigned graphical interface UI, based on a modern reactive Javascript framework, which allows users to perform complex searches, view detailed quality reports and visualize/order data.

### 4.4. ARM DOI

**Figure 8:**
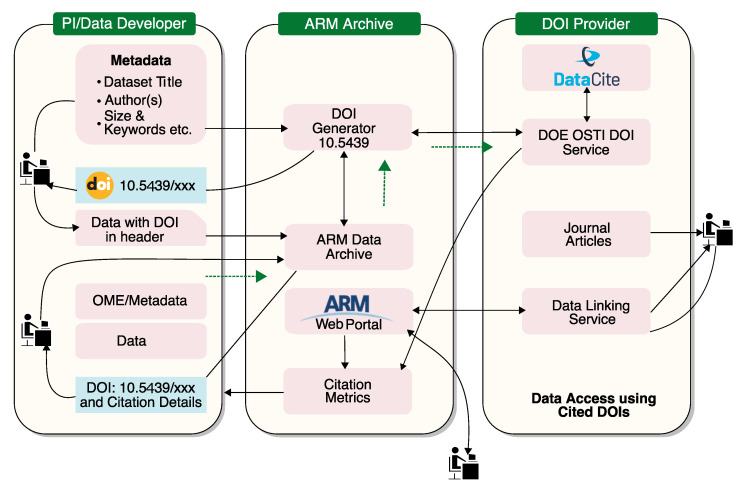
ARM DOI assignment workflow (source: ARM).

Regarding the data citation system, a scalable architecture [70] for scientific data was implemented for Big Data level data. This architecture is based on Digital Object Identifiers (DOIs) to facilitate the citation of datasets. This makes it easier for users to find the exact data in articles. These DOIs are assigned to an ARM dataset product so that DOIs can be managed dynamically. Figure 8 shows a scalable architecture for data citation.

### 4.5. DQ Tools

Web application technologies evolve rapidly with continuous innovations and improvements. This paper focuses on the popular Spring Boot Java-based framework for building web and enterprise applications and on how it provides flexibility for a service-oriented architecture. One challenge with any Spring-based application is the level of complexity in configurations. Spring Boot makes it easy to create and deploy standalone, production-grade Spring applications with minimal Spring configuration. For example, if we consider a Spring Model View-Controller framework, we need to configure a dispatcher servlet, web jars, a view resolver, and a component scan, among other things. To solve this, Spring Boot provides several auto-configuration options to set up the application with all the required dependencies. Another challenge is determining the framework dependencies and associated library versions required to develop a web application. Spring Boot provides easy dependency management using a comprehensive, flexible framework and related libraries in a single dependency that provides all the Spring-related technology needed for entry-level projects compared to CRUD web applications.

This framework provides several additional features used in different projects, such as an embedded server, security, metrics, health checks, and externalized configuration. Web applications are usually packaged as a war file and deployed to a web server. However, the Spring Boot application can be packaged as either a war or jar file, allowing the application to run on the application server without installation or configuration.

The application consists of three main components: a front-end UI form (for collecting user-submitted data), an API (which accepts HTTP/S requests either through a UI form or a command call, such as curl or Postman), and the database (where the data is stored). The UI is a simple form with a series of fields describing the data quality problem for the data in question. After submitting the form, the data is validated (checked for required fields and expected formats). If the data is valid, it is sent as a JavaScript Object Notation (JSON) request via a resource URL to the REST API.

### 4.6. Data Storage

**Figure 9:**
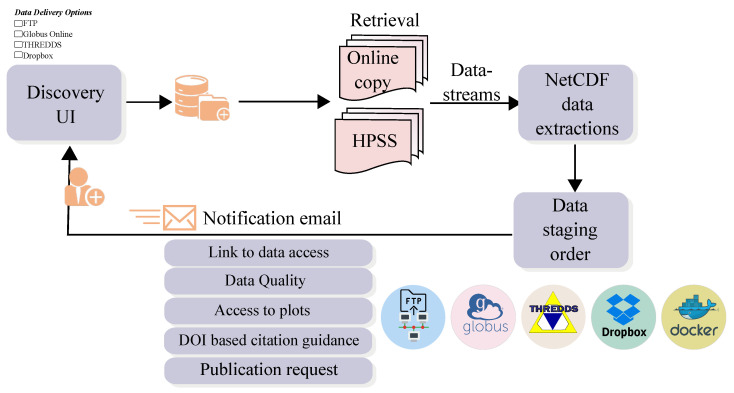
ARM workflow: data retrieval, packaging, and delivery (source: ARM).

The ARM Data Center uses a reliable, fast, multi-tiered high-performance storage infrastructure. It provides multiple methods and protocols for data access and downloads, such as FTP, GridFTP, real-time web services, and the THREDDS data server. When a user places an order through the data discovery tool, the data is retrieved from either the high performance storage system or online storage and made available for download. An email notification is then sent to the user with all the appropriate download options. The email also contains information on data quality, related publications, and how to cite the data. Storing the data on hard drives allows quick and easy access to the data, but, due to the sheer volume of data from ARM, storing all of the data on our online disk storage is expensive. ARM stores approximately 750 TB of data on online disk storage and an additional 2.2 PB (as of August 2020) on Oak Ridge National Laboratory’s High Performance Storage System. Based on the user’s data ordering request, we use programmatic retrieval of files from either the online disk storage or the High Performance Storage System. Figure 9 shows the ARM workflow.

### 4.7. Data Distribution

ARM data can be accessed via the RESTFul web service (https://adc.arm.gov/armlive, accessed on 10 January 2022). Due to increased field campaigns and high-resolution model outputs, the volume of data is expected to increase five-fold in the next 5 to 10 years. Given the projected growth and expanding user community, ARM must continue to learn and incorporate innovative data and computing capabilities for its users.

This interface allows users to query data from ARM directly in their workspace and automate machine-to-machine downloads. A web service for downloading is prevalent, especially for continuous real-time data and repeat orders.

## 5. Partial Results and Discussions

**Figure 10:**
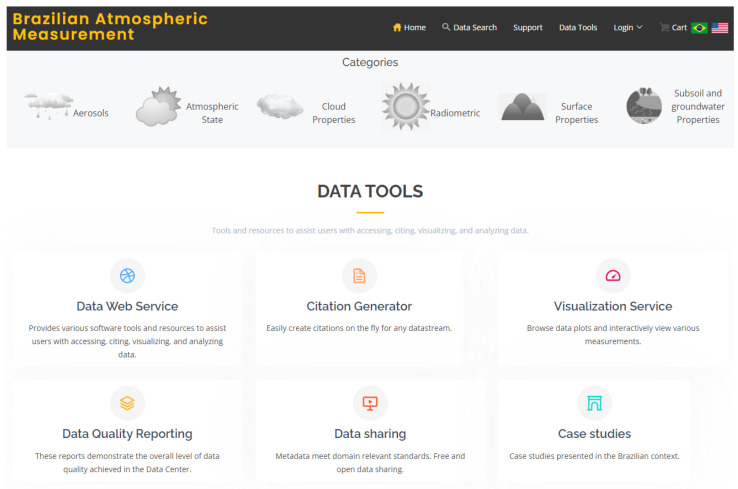
Data portal interface for Publishing and Delivery of Open Data.

A preliminary data portal version was implemented, based on the topics raised in the previous sections. Figure 10 presents the design of the main interface with the characteristics of the portal. This version highlights the categorization and cataloging of environmental data products for their availability. The design was based on FAIR principles.

**Figure 11:**
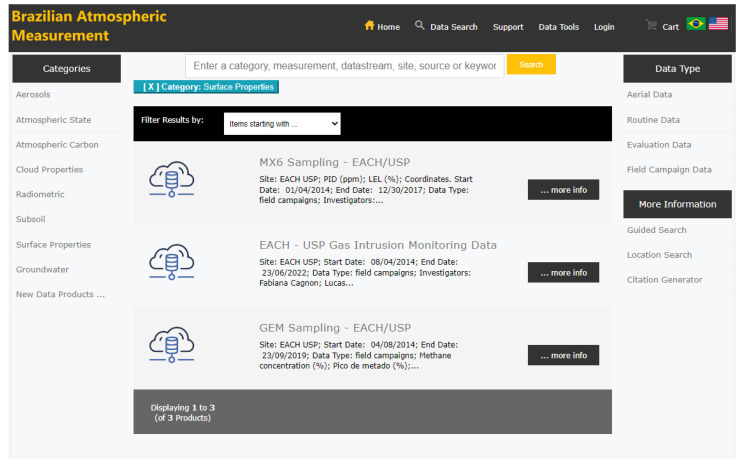
List of data fetched in the Data Discovery interface.

The Findability principle can be applied using a data discovery tool to locate the data easily. Data products have a global identifier and rich metadata, such as measurement range, creation date, temporal resolution, license, file type and data volume (GB). Our findability architecture has three relevant components: (1) a metadata collection system; (2) an indexing system to build the distributed search index for millions of data files; and (3) a graphical user interface, based on UX/UI studies carried out by ARM (Figure 11 shows a searched data list interface). This allows users to perform complex searches, obtain detailed quality reports, and visualize and download data.

It is worth mentioning there are other components that are portal requirements and must be implemented. One example is the citation system. Based on ARM experience, data products can be uniquely identified by digital object identifiers (DOIs). Their metadata can be exported to different types of standards, such as International Organization for Standardization (ISO) 19115-2, the notation JavaScript Object Standard for Linked Data (JSON-LD) and the Content Standard for Digital Geospatial Metadata (CSDGM). This allows the metadata to be shared with other data centers, such as Google Dataset and NASA/EARTHDATA. This component will be implemented in the next version.

The Accessibility principle can be applied by establishing a trusted repository system. We do this through policies established in our metadata system. For example, it has reliable, fast, and multi-tiered high-performance storage resources at the infrastructural level. The data access and download methods are through real-time web services, FTP protocol, GridFTP protocol and the THREDDS data server.

Interoperability is achieved through an established language for metadata based on standards. The data model (data format) used for management is NetCDF. This model is used to represent/store large volumes of multidimensional data. Its use is vital in storing data from sensors (heterogeneous captured data) and when data captures are periodic.

The Reusability principle can be applied by maintaining licenses that guarantee the integrity of the data (e.g., origin and accurate information). The tools are, thus, made available to the scientific community for free access.

The source code from the preliminary version of the Data Portal is available on GitHub (https://github.com/encinasquille/ProjSGA.git, accessed on 15 January 2022). As regards the next steps for implementing the data portal, we look forward to storing data from different Brazilian data sources. Integration with other portals and cloud environments, such as Amazon Web Services (AWS), will make the data portal more flexible.

The portal architecture presented was designed to process and analyze large volumes of environmental data in the Brazilian context. The architecture also allows integration with other portals, such as SiBBr, which integrates spatial data from more than 140 Brazilian biodiversity institutions. However, several research efforts generate or collect thousands of data in the atmospheric field daily. A clear example is the GoAmazon project, which collects data on aerosols from the Brazilian Amazon, but does not have a data portal for its dissemination. In addition, Brazilian institutions, such as INPE (Instituto Nacional de Pesquisas Espaciais [64], already provide open data with minimum data quality standards for reuse through its main portal. This data provides essential information for analysis and decision-making.

We can point out some limitations regarding the current data portal version, which are related to future work challenges.

- Scattered data: As mentioned before, Brazilian atmospheric data are scattered in many different data portals and repositories. We need to partner with different Brazilian research institutions to centralize this data in our portal;
- Data curation: We need to develop a data curation scheme, or something similar, to ensure the quality of the data submitted;
- Costs: Since data increases exponentially, the data portal maintenance costs should grow in the coming years. Even though the architecture is flexible, maintenance resources will be necessary, and financial support from various institutions may be needed.

## 6. Conclusions

This work presented a scalable data portal architecture and a preliminary version of the portal. The architecture proposed delivers open data, allows processing of Big Data, and provides tools that help users catalog, publish and analyze environmental data. This architecture practices FAIR principles to manage knowledge by integrating and reusing published scientific data. These principles enable long-term maintenance of digital assets. Data management is a challenge to the scientific community and must be overcome. In this context, the architecture developed was based on the experience of the Atmospheric Radiation Measurement Center, which presents specific tools that enable optimal management of the various national and international climate research measurements. The tools for data centers discussed at ARM are metadata management, data discovery, data citation, data access via web services, and data quality system.

For data processing, we propose an architecture under an elastic infrastructure according to the amount and complexity of the data, which can be processed in a public or hybrid cloud. It offers processing strategies depending on the context, in-memory processing with the use of Spark, on-disk as Hadoop and other alternatives such as GPUs or RAPIDs. To maintain elasticity, we considered a model distributed across microservices and containers to guarantee scalability. The database management system proposed is a hybrid model. For Big Data, we considered NoSQL, due to its distributed architecture, SQL in some tools, due to its relational structure as a metadata system, and other alternatives, such as GraphQL or Hbase. This architecture allows publishing and provision of open environmental data, as well as data preparation through query engines, artificial intelligence models, data analysis and visualization through APIs.

This work demonstrates that interpreting the FAIR principles for implementation with Big Data support and a specific domain is complex. Some implementations gave solutions, and these can thus be reused. However, there are data management issues regarding ensuring quality throughout the data life cycle. With this proposal, we seek to encourage good research practices concerning multiple types of environmental data. This could allow various users (scientific community, analysts, decision-makers and the general public) to participate from anywhere, giving rise to a ubiquitous solution. Finally, open science practices are beneficial to continue discovering knowledge openly and collaboratively.

## Figures and Tables

**Table 1:** Comparison of open data portals according to the FAIR principles.

Data Portal	Big Data	Findability:	Accessibility:	Interoperability:	Reusability:
		F1, F2, F3 & F4	A1, A1.1, A1.2 & A2	I1, I2 & I3	R1, R1.1, R1.2 & R1.3
INPE [64]	✘	F2	A1, A1.1.	I1	R1, R1.1
SiBBr [47]	✘	F2	A1, A1.1.	I1	R1, R1.1
Linked Open Data [65]	✘	F1, F2, F3 & F4	A1, A1.1 & A1.2	I1, I2 & I3	R1, R1.1, R1.2 & R1.3
ALA [66]	✔	F1, F2, F3 & F4	A1, A1.1, A1.2 & A2	I1, I2 & I3	R1, R1.1, R1.2 & R1.3
GBIF [67]	✔	F1, F2, F3 & F4	A1, A1.1, A1.2 & A2	I1, I2 & I3	R1, R1.1, R1.2 & R1.3
LINCS [68]	✔	F1, F2, F3 & F4	A1, A1.1, A1.2 & A2	I1, I2 & I3	R1, R1.1, R1.2 & R1.3
ARM [69]	✔	F1, F2, F3 & F4	A1, A1.1, A1.2 & A2	I1, I2 & I3	R1, R1.1, R1.2 & R1.3
Proposed architecture	✔	F1, F2, F3 & F4	A1, A1.1, A1.2 & A2	I1, I2 & I3	R1, R1.1, R1.2 & R1.3
